# Adrenalin in Asthma

**Published:** 1911-02-11

**Authors:** J. Johnstone Jervis

**Affiliations:** Assistant Medical Superintendent. The Infirmary, Park Hill, Dartmouth


### Editor's Letter-Box.

### ADRENALIN IN ASTHMA.

Sir,—I was much interested to read the note in last week's issue of The Hospital on the use of adrenalin in the treatment of asthma.

My experience of the value of the drug in these cases coincides with that of the writer. I have used it as a routine treatment for some time now, and have found it almost uniformly successful in cutting short the attack and bringing speedy relief to the patient.

It seems to act directly upon the vessels in the walls of the small bronchial tubes, causing them to contract and increasing their tone, thus relieving congestion and giving the patient more airway.

But asthma is not the only pulmonary condition in which I have found the administration of adrenalin beneficial. In bad cases of chronic bronchitis with well-marked emphysema, where the patient is the subject of recurring attacks of coughing accompanied by dyspnœa, adrenalin (given hypodermically) has often proved itself of immense value in arresting the cough and restoring the breathing to a more normal state. At the same time it seems to decrease the amount of bronchial secretion, though if that be very copious, as in such cases it generally is, this action is not sufficient. In that event I find that by combining it with atropine one gets very satisfactory results.

I have also used it in cases of cardiac asthma where the blood-pressure has been low and cardiac compensation failing, but here the good effect has not been so marked as in the other conditions mentioned.

Whilst using the drug one has to keep a very close watch upon the heart, which is apt to become overtaxed if too much be given. In whatever condition it is given it always adds to the heart's work, but it equally adds to the tone of the whole vascular system and so enables the heart to overtake that extra work without risk of over-exertion. Arterio-sclerosis, chronic Bright's disease, and aortic disease in my opinion absolutely contra-indicate its use, as in these cases the blood-pressure is quite high enough without adding thereto.

Adrenalin as a pulmonary remedy is without a doubt of very great value and a distinct asset to the physician's pharmacopœia.—I remain, yours faithfully,

J. Johnstone Jervis, M.B., Ch.B.Edin.,
Assistant Medical Superintendent.

The Infirmary, Park Hill, Dartmouth, January 31.

